# ‘Fighting an uphill battle’: a qualitative study of the challenges encountered by pharmacy workers when providing services to men who have sex with men in Dar es Salaam, Tanzania

**DOI:** 10.1080/16549716.2020.1770985

**Published:** 2020-06-08

**Authors:** Joakim Öhman, Markus Larsson, John Kashiha, Anette Agardh

**Affiliations:** a Social Medicine and Global Health, Department of Clinical Sciences, Lund University, Malmö, Sweden; b Community Health Education Services & Advocacy (CHESA), Dar es Salaam, Tanzania

**Keywords:** MSM, pharmacies, Tanzania, discrimination, STI, HIV

## Abstract

**Background:**

Previous research suggests that Tanzanian MSM might prefer consulting pharmacies and drugstores, rather than public healthcare services, when in need of STI medicines and treatment. Yet, few studies have explored the experiences of providing services to MSM clients among those working at pharmacies and drugstores and examined what challenges they encounter in providing these services.

**Objective:**

To gain increased knowledge and understanding of the perceived challenges encountered by pharmacists and drugstore workers when providing STI services to MSM clients in Dar es Salaam, Tanzania.

**Method:**

In early 2016, 16 semi-structured interviews were conducted with persons working at private pharmacies and drugstores in Dar es Salaam. Data were interpreted through qualitative content analysis.

**Results:**

The overarching theme that emerged was labelled ‘Fighting an uphill battle’, which reflected the challenges pharmacy workers experienced during interactions with MSM clients, and in particular service provision. Pharmacy workers tried to act upon the best of their knowledge to meet the needs of clients, given their understanding of risks and obstacles that MSM faced. Yet, the lack of educational and professional preparedness and insufficient financial and human resources, regarded as necessary to meet the needs of a stigmatised client group, formed barriers for effective service delivery.

**Conclusions:**

In order to support pharmacists and drug-store workers in Tanzania to address perceived challenges for service delivery to MSM clients, systematic and continuous training on MSM’s sexual health is required. *Furthermore, inter-professional cooperation that harnesses provider involvement from all tiers in the healthcare system is essential* to offer complementary services to ensure proper STI care and treatment. Thus, interventions that focus on *inter-professional* communication and interaction between pharmacists and physicians could have a positive impact on timely referrals of suspected STI cases among marginalised populations.

## Background

Worldwide, men who have sex with men (MSM) constitute a high-risk population for HIV and other sexually transmitted infections (STIs), and reports demonstrate that the HIV incidence continues to increase in several regions in this population [1]. According to the UNAIDS Strategy 2016–2021, HIV prevalence among MSM is among the highest in Africa: 15% in Western and Central Africa and 14% in Eastern and Southern Africa [2,3]. In Tanzania, studies have shown that the HIV prevalence in MSM is four to five times higher than in the general male population [4,5]. Additionally, a number of studies in Tanzania, and elsewhere in Sub-Saharan Africa, have reported high-risk sexual behaviours, such as limited condom use and multiple concurrent partnerships in this population, which further exacerbate the risk of HIV and STI transmission [4,6–10].

In the majority of African countries, homosexual acts are illegal, including in Tanzania with up to lifetime imprisonment for sexual acts between men [11,12]. Apart from legal barriers, traditional societal and cultural values and norms around sexuality also influence the behaviours and characteristics that are deemed acceptable in a context [13]. Individuals and groups that deviate from these accepted behaviours and characteristics, for example by engaging in homosexual relationships, run the risk of becoming stigmatised, which refers to some kind of punitive measure taken by the collective, such as discrimination and marginalisation [14,15]. The linkages between homosexual stigma, the societal disapproval of homosexuality [16], and hostile attitudes against MSM in the healthcare system have previously been investigated across the Sub-Saharan African context [17–19]. In Tanzania, studies have revealed that the stigma against homosexuality form both actual and perceived barriers to access HIV related services for MSM and has a discouraging effect on their healthcare seeking behaviour [20–22].

Pharmacies and drugstores have a central role in community health and are fairly accessible compared to hospitals and clinics that tend to be overcrowded and costly in many low-income countries [23]. Evidence suggests that Tanzanian MSM might prefer consulting pharmacies and drugstores, rather than public healthcare services when in need of STI medicines and treatment [22]. One explanation seems to be that drugs are more readily available to MSM without having to disclose private and sensitive information that potentially could reveal their sexual history, for example the source of infection. However, problems related to illegal dispensing and stocking of prescription drugs, unqualified staff and absence of valid permits have been documented among pharmacies and drugstores in Tanzania [24–26].

Nevertheless, employees at pharmacies and drugstores appear to play an important role for MSM’s healthcare, in Tanzania and elsewhere, and might therefore influence the health of the MSM population [22,27–29]. In a previous study in Tanzania, we found that the emergence of relationships between pharmacists and drugstore workers and MSM clients facilitated the process of STI service provision [27]. As pharmacists and drugstore employees gradually became familiarised with the complex issues that MSM clients dealt with, they also became increasingly engaged in service provision. At the same time, the findings further revealed internal conflicts, caused by the service providers’ personal beliefs around homosexuality and ambivalent feelings of involving MSM in service delivery – possibly related to their insufficient knowledge of gender identity and sexual orientation, and MSM clients’ specific needs [27]. Consequently, there is a need for further research that seeks to understand the challenges this professional group faces regarding service provision to MSM clients. Such knowledge would inform community health programmes that seek to improve the care of marginalised and vulnerable groups. Thus, the aim of this study was to gain increased knowledge and understanding of the perceived challenges encountered by pharmacists and drugstore workers when providing STI services to MSM clients in Dar es Salaam, Tanzania.

## Method

### Study design

A qualitative methodological approach was chosen for the study. Here, focus is placed on acquiring an in-depth understanding of the participant’s perspectives and subjective experience of a specific issue or phenomenon [30]. This approach was deemed as the most suitable for gaining access to in-depth and rich descriptions of pharmacy and drugstore workers’ experiences of the perceived challenges associated with service provision to MSM clients.

### Study setting

In early 2016, 16 semi-structured interviews were conducted with persons working at private pharmacies and drugstores in Dar es Salaam, Tanzania. Dar es Salaam is the largest city in Tanzania, with a population of more than four million [31]. Albeit not the capital city, it is the country’s financial and commercial hub. In Tanzania, the Tanzania Food and Drugs Authority (TFDA) holds the responsibility for pharmaceutical regulations [32]. There are two types of drug-dispensing outlets – part I pharmacies (retail pharmacies) and part II pharmacies (drugstores, locally known as ‘Duka la Dawa Baridi’). Part I pharmacies are licensed to dispense both prescription-only medicines (POMs) as well as over-the-counter (OTC) medicines, and are required to be operated by a registered pharmacist. Part II pharmacies are by regulation only allowed to sell OTC medicines, and storeowners do not necessarily have to possess specific pharmaceutical qualifications. However, it is compulsory for all staff members interacting with customers to have basic medical knowledge [24,25,33].

Due to problems of maintaining regulations and illegal sales of POMs at part II pharmacies, TFDA introduced the accredited drug dispensing outlet (ADDO) programme in an effort to address the situation [25,34]. The ADDO programme focused on altering the expectations and behaviour of those who own, utilize or work at part II pharmacies through education, training and supervision. Accredited part II pharmacies are authorized to dispense a limited range of essential POMs, including some antimicrobials, and in 2013 there were approximately 9000 accredited (or awaiting accreditation) part II pharmacies [34,35].

### Study participants and ethical considerations

This study was approved by the Senate Research and Publication Committee at Muhimbili University of Health and Allied Sciences, Tanzania (ref. no. 2015–11/AEC/Vol.IX/102). The 16 participants were recruited with help of a local community-based organisation working with key populations, such as MSM. In order to be eligible for the study, participants were required to a) be over the age of 18 years, b) be available for an interview during the time of data collection, c) work at a part I or part II pharmacy, and d) have previous experience of providing services to MSM clients. Due to the fact that participants worked at both part I and part II pharmacies, they are hereafter referred to as *pharmacy workers*. The community-based organisation provided the research team with a list of pharmacy workers in Dar es Salaam who met the inclusion criteria. During a consultative process with the organisation, the research team selected participants based on their ability to offer a private place for the interview and on their geographical distribution. A letter explaining the study purpose and requesting for an interview was given to the selected participants. Out of 20 contacted, 16 responded back to the research team with an interest to participate in the study. Once contact had been established, the participant was asked to suggest a time for the interview. All interviews were conducted in private, either at the workplace, or at a locality nearby. At the time of the interview, the participant received a written information letter that stated study purpose, explained that participation was voluntary and anonymous, that they could refuse to answer the questions, and interrupt the interview at any time. This letter was given in both English and Swahili (the official national language of Tanzania). Before starting the interview, informed verbal consent was taken by asking the participant whether they wanted to continue the interview after reading the information letter. Verbal consent was chosen to ensure anonymous participation, considering the potential controversial nature of the topic. All pharmacy workers agreed to participate; thus, the total number of study participants was 16. No compensation was provided to the participants for the interview.

### Data collection

The interviews were conducted by ML and AA. All interviews were audio-recorded and transcribed verbatim by the same person. The interview duration ranged between 50 minutes and one and half hours. During the interview, questions were asked in an open-ended manner to encourage discussion and rich information. A pre-tested interview guide was used, covering a wide range of topics from attitudes towards same-sex conduct to service-provision to MSM clients. The interviews were conducted in English; however, an interpreter from the community-based organisation was always present in order to provide translation to Swahili if needed. Four participants required this assistance, of which two only for some of the responses. For interview text transcription, the same interpreter who was present during the interview translated the passages that had been given in Swahili into English. Saturation was considered to have been achieved when no further significant information emerged from additional interviews [36], and both interviewers deemed this accomplished after the 16th interview. Additional information about data collection and procedures has previously been presented in an earlier article using the same dataset [27].

### Data analysis

The data were analysed by the first author (JÖ) according to the qualitative content analysis approach, as described by Graneheim and Lundman [37]. The use of this analytical method allows for interpretation of the apparent and visible components of the text, e.g. the manifest content, as well as the underlying meaning of the text, e.g. the latent content. As a first step, the 16 transcribed interviews were read repeatedly by all authors to obtain a comprehensive understanding of the content. Following this, the interview text sections that dealt with issues relating to the aim were extracted and compiled into segments, which constituted the units of analysis. The segments were then divided into meaning units (an arrangement of words, sentences or paragraphs, containing aspects associated with each other). The meaning units were condensed and labelled with codes. Next, based on similarities and disparities, codes were clustered together into categories, which comprised the manifest content. As a last step, an analysis of the latent content, or the underlying meaning and patterns of the categories, was conducted and resulted in one overarching theme and three sub-themes. All authors were involved in the various stages of the analysis, and consensus concerning categories and themes was arrived at through mutual discussions.

## Results

Table 1 presents the socio-demographic characteristics of the 16 participants. Most participants were female, in their twenties or thirties. The majority had a professional background in either nursing or pharmacology. The proportion of participants who worked at part I and part II pharmacies, respectively, was equal.

**Table 1.** Participant characteristics.

Participant	Sex	Age	Professional background	Place of work
1	Female	28	Nurse (owner)	Drug store
2	Female	34	Pharmacy assistant	Pharmacy
3	Female	35	Nurse	Drug store
4	Female	29	Pharmacy assistant	Pharmacy
5	Male	26	Pharmacist	Pharmacy
6	Male	45	Pharmacist technician	Pharmacy
7	Female	33	Nurse	Pharmacy
8	Male	53	Teacher (owner)	Drug store
9	Female	29	Nurse	Drug store
10	Female	38	Nurse assistant (owner)	Drug store
11	Female	38	Medical doctor (owner)	Drug store
12	Female	26	Nurse (owner)	Drug store
13	Female	48	Pharmacist	Pharmacy
14	Female	32	Medical assistant (owner)	Drug store
15	Female	27	Nurse	Pharmacy
16	Female	41	Pharmacist (owner)	Pharmacy

**Figure 1.**
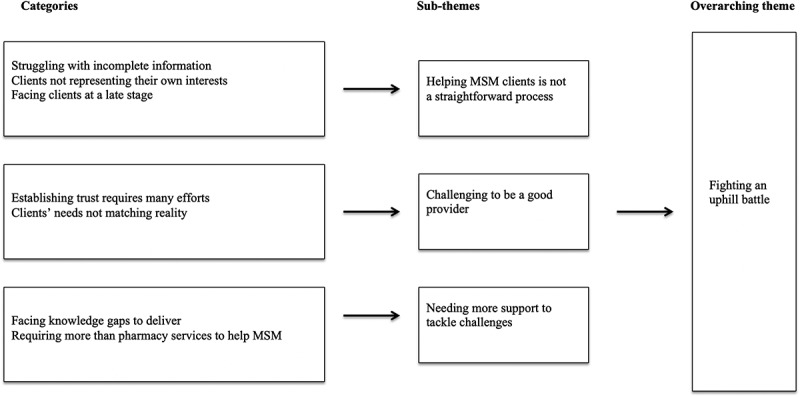
Schematic figure of the analysis of pharmacy workers’ perceived challenges of providing STI services to MSM clients in Tanzania.

The qualitative content analysis of the 16 interviews resulted in an overarching theme: *Fighting an uphill battle*. This theme described the challenges pharmacy workers experienced in their interaction with MSM clients, and in particular service provision. Pharmacy workers tried to act upon the best of their knowledge to meet the needs of clients, given their understanding of risks and obstacles that MSM faced. Yet, pharmacy workers experienced a lack of educational and professional preparedness to deal with MSM clients and their challenges, stemming from the broader homosexual societal stigma. Coupled to this was a realisation that they were unable to access sufficient financial and human resources that participants regarded as necessary to meet the needs of a stigmatised client group. Altogether, this formed barriers for effective service delivery. As shown in Figure 1, the overarching theme consists of three underlying sub-themes: (I) *Helping MSM clients is not a straightforward process*, (II) *Challenging to be a good provider*, and (III) *Needing more support to tackle challenges*. Each sub-theme illuminates an aspect of the perceived challenges that pharmacy workers in Tanzania faced when dealing with MSM clients, and the sub-themes consist of different categories (italicized and underlined). Schematic figure of the analysis of pharmacy workers’ perceived challenges of providing STI services to MSM clients in Tanzania.

### Helping MSM clients is not a straightforward process

All pharmacy workers indicated that they had served MSM clients with STI related issues. They complained, however, about the absence of complete and reliable information regarding the clients’ sexual history and symptoms, which they felt hindered them in their role and function as pharmacy workers. In comparison with other clients, MSM clients were perceived as more evasive and difficult to assist.

#### Struggling with incomplete information

MSM clients were described as insecure and imprecise in their interaction with pharmacy workers, and clients’ inability to explain their symptoms or problems was a challenge.

One of the biggest challenges for me is that most of the MSM, when it comes to explaining what they are suffering from, they cannot. When they come, they do not find what words they should use to tell me their problems. (Participant 13)

The challenges of eliciting reliable responses complicated treatment provision, as pharmacy workers had limited information about the problem and possible causes when trying to make a diagnosis.

They say that they do not know what caused the pain. Some can even say that they have pain in the head and then ask for a specific drug that they want, often some kind of antibiotics. It makes it difficult for me to know what to do. (Participant 3)

The evasiveness among MSM contributed to a feeling of not being able to reach out to the clients and formed a barrier to open and direct interaction.

*Clients not representing their own interests* was another aspect, e.g. *‘Sometimes they send a friend, someone who is open: “You know it feels this and this and I need that and that”’* (participant 8), and the narratives offered vivid details of encounters with clients who acted on behalf of someone else, as illuminated by the statement below.

I remember one client who came with a note and said, “Read this message” and it had a name of a drug. So, I asked him, “What kind of disease are you suffering from?” But he did not say. I understood that this client did not know what type of disease he was suffering from, so I tried to ask him, “Who are sending you here to buy this medicine?” and he said that a friend had asked him to go. After long time when we had talked and I said, “Do not be scared, this is a secret. That man who sent you, who is he?” So, he said that it was not his husband but like a friend. So, they were already having sex, you see, so that man he was HIV positive and asked this client to go and buy a medicine. (Participant 10)

On the one hand, pharmacy workers could understand the reasons why some clients were reluctant to come forward, as they wanted to avoid disclosing personal information that potentially could expose them as MSM. On the other hand, this complicated trust building since it was difficult to determine whether a client represented himself or someone else. The following quotation serves as an illustration of how this problem was tackled:

First of all, you cannot always tell if it is a friend. So, you help them. But if I know, I say, “[name], go and tell that friend of yours that he should come here, I will not hurt him.” (Participant 8)

MSM clients often approached pharmacy workers when STI symptoms already had worsened and *facing clients at a late stage* was frequently mentioned.

In most of the cases MSM are coming with a serious infection, like having wounds in their back [rectum], and their back is really bad; the infection is much spread. It has to be treated very quick. (Participant 2)

When asked about problems, MSM clients refused to explain the source of the symptom, e.g. *‘Denial of infection, for example when there is pus from the rectum they say, “I did not do anything, I found it getting out from there”’* (Participant 15). These types of distracting manoeuvres were thought to stem from the negative climate surrounding homosexuality in Tanzania, and participants believed self-stigma was a reason why MSM had not sought treatment earlier.

A big problem is for the community to accept these people and those MSM they do not accept themselves. It is like they think, “I am an MSM and they will not treat me well”. (Participant 6)

### Challenging to be a good provider

The theme ‘Challenging to be a good provider’ reflected a dilemma confronted by pharmacy workers during service provision to MSM clients. Pharmacy workers tried to make sense of the complex situation these clients faced, and the insights gained drew attention to the specific efforts, service assistance, that MSM needed because of their experience of stigma and discrimination. Yet, when considering the reality of the stressful environment of pharmacy work, a discrepancy emerged regarding perceived expectations of clients and own ability to deliver.

#### Establishing trust requires many efforts

Participants pondered the issue of trust, a prerequisite for successfully engaging with MSM clients, who seemed fearful of the reactions of providers. Time and being creative were stressed as particularly crucial.

You need to be creative by being there for them, and you need time. One time I met this boy, maybe in his young twenties. At first, he was so shy. He told me that he had abdominal pain and wanted some amoxicillin. At that point there were few customers in my store, so I asked him, “Amoxicillin? That is not for your stomach, tell me what is wrong with you?” He hesitated but I remained quiet and after a while he said that it was for his penis. He had discharge and pain when he urinated. So, I asked him for how long he had suffered from this problem and the possible reasons. He still hesitated so I told him that we have many young men in this store with penile problems and that he shall not fear anything. Then he told me he had had anal sex with another man but was afraid of going to the doctor. He thought that someone could see him at the clinic and report back home. So, I helped him and gave him the drugs after he described the symptoms in more detail for me. (Participant 6)

Yet, the demands on staff time and the ability to provide privacy were not perceived as compatible with the fast-paced environment that characterised pharmacies and drugstores, reflected in the category *clients’ needs not matching reality*.

You see, I have worked here for almost eight years and a lesson learned is to let him be open and you are friendly to them, then the truth will come. But you need time. That is the issue. And here, this place is busy. It is difficult for me to spend time with them. (Participant 2)

Participants thought of various solutions for privacy and delineated steps that should be taken to offer discretion, such as using an adjacent room for consultation. However, a common situation was being the sole staff member on duty, which restricted possibilities for privacy.

Yes, we have a room. But for me to go there I must close the shop. It is just me working here you know. So, I never use it. (Participant 4)

Others complained that the set-up of their pharmacy or drugstore did not allow privacy in the first place, which acted as a deterrent for confidential consultations.

But at my pharmacy there is hardly any privacy. It is not like at the private pharmacies when someone can get inside in a room and talk privately. Here it is like open. So, it will be difficult. The situation does not allow me to talk privately very much. (Participant 2)

The insight about the time-consuming efforts required to create trust and privacy left pharmacy workers with a feeling of being inadequate, e.g. *‘I do not know what to do sometimes. How can I be there for them* [MSM clients]*?’* (Participant 6). Others resorted to rational explanations, referring to their profession as pharmacists or drugstore workers – not a doctor, e.g. *‘I can only do what I can. The rest is up the client to find out at the hospital’* (Participant 1).

### Needing more support to tackle challenges

Pharmacists’ own narrow understanding of MSM and the MSM community, and limited existing support infrastructure in the healthcare system, emerged as significant barriers to effectively address the health needs of the MSM population. Due to the socially disadvantaged status of MSM as an ‘outsider group’, pharmacy workers experienced difficulties to connect with their problems and needs, resulting in miscommunication and tensions. Moreover, being a pharmacy worker was concomitant with certain constraints concerning the type of services that could be given, and in order to offer MSM clients a comprehensive care approach, beyond pharmaceutical services only, a need for coordination with MSM-sensitised clinicians was emphasised.

#### Facing knowledge gaps to deliver

Being sensitised on MSM’s sexual health was crucial in order to provide adequate services. Participants reflected upon their educational needs and expressed a desire to develop their capacity, e.g. *‘I think that if I get more knowledge about their behaviours and problems, I would be able to help them better because we have a lot of MSM here’* (participant 2). Pharmacy workers believed that the impact of an unskilled workforce would have detrimental consequences for MSM, e.g. *‘Lack of knowledge is also a problem, we do not have knowledge to save the group* [MSM]*’* (participant 14). Insufficient understanding of MSM could result in conflicts with the client, as evidenced in the following statement:

I was abused by one of the MSM because I did not understand what he needed. So, he shouted and talked loud to me. It has actually happened to me two times. It is also a problem to understand this community: their characters, their behaviours and so on. In my college we never talked about key populations. (Participant 15)

As shown by the above statement, pharmacy workers felt that they lacked knowledge about MSM’s situation and health needs. While participants logically could make sense of clients’ frustration, more knowledge was required to address the needs of this population. However, some pharmacy workers had identified ways in which they could strengthen their knowledge of MSM.

I studied one good course that helped me. It is a topic through the community studies. It talks about how you can cope with the community. It made me realize that there are differences in people and that is like normal. It is just a short course you take as an employee when you want to add more knowledge. More of us [pharmacy workers] should attend this course. (Participant 12)

Participants’ comments about effectively addressing the health needs of MSM revealed structural barriers and emphasised the lack of coordination with sensitised providers. The category *requiring more than pharmacy services to help MSM* captured the constraints related to what participants, as pharmacy workers, could do for their clients, and the call for integrated and specialised services that MSM could use.

I think that they should be able to go to the hospitals and get treatment. They need to be investigated. Of course, I can provide the first … like the first aid … but they also need to go to hospitals and be tested. (Participant 10)

Participants alluded to the importance of availing MSM opportunities to access certain services, such as laboratory testing, which stressed the involvement of the broader health system.

The most important thing is to educate healthcare workers to be able to help them. There are so many that do not accept MSM. (Participant 2)

A need to be better connected with MSM-sensitised healthcare workers was expressed, and participants believed that clients could be referred for testing and diagnosis, if they were confident that clients would be treated well at the clinic or hospital.

There must be a specific, trained, person, who I refer to at a centre. Someone who knows about them. If I just send him to an ordinary doctor he will not go there. (Participant 16)

Participants who had already established connections with a place that provided MSM friendly services were relieved to have this system in place.

I refer them to [name]. I have talked with them before and they are friendly to MSM, so I think it is a good place. It feels right to refer them there. (Participant 3)

## Discussion

The findings from this study uncovered unique narratives that described the challenges of providing STI services to MSM clients, as experienced by pharmacy workers in Dar es Salaam, Tanzania. One overarching theme and three sub-themes were identified from the responses of the participants. The overarching theme, *Fighting an uphill battle*, captured the difficulties pharmacy workers endured when dealing with a group marked by stigma and was a metaphor for the participants’ approach towards overcoming the sometimes daunting task of providing services to this population. These findings complement our previous study [27] by focusing on the challenges that pharmacy workers encountered. While the bonds between pharmacy workers and MSM clients facilitated service provision [27], these new findings enhance our understanding of factors that may have an impact on sensitised and efficient pharmaceutical service delivery, i.e. counselling, drug treatment and referral. Most notably, the new findings revealed a lack of professional preparedness to deal with MSM clients and their needs. Taken together with the previous results [27], these findings contribute to a more holistic understanding of pharmacy provision of health services to a stigmatised group.

Pharmacists and drugstore workers belong to a group of healthcare providers who are highly accessible to the public through their role of administering drugs and providing advice and guidance to clients [38]. With a ubiquitous presence in the community, they are in a unique position to counsel customers with sexual health advice, and to refer them to relevant healthcare services [39]. Our findings suggest that pharmacists and drugstore workers indeed assisted MSM clients with their health issues, mainly about STI related problems, and provided advice to clients regarding their treatment needs, as well as administered drugs. Consequently, a main finding from this study is that pharmacy workers are an important access point to healthcare for MSM, which also has been confirmed by a previous study from Tanzania focusing on the MSM population itself [28]. Agardh et al. in their qualitative study of perceptions and experiences of using pharmacies and drugstores for sexual health and STI problems, found that the possibility to choose a provider that seemed friendly was an important care enabler for MSM [28]. MSM friendly care, e.g. non-judgemental and sensitised providers, has been identified as a crucial incentive for health-seeking among MSM in different settings [40]. Our data indicated that allocating time and engaging in dialogue with clients were important factors for building trust, which stresses the role of the relationship between the provider and client. As highlighted by Bonner, the long-term relationship that evolves in pharmacy care carries a unique opportunity to establish trust and confidentiality, thereby potentially removing deterrents related to disclosure of sexual history [41].

Yet, the interviews revealed significant barriers to elicit reliable information from clients, and participants experienced both confusion and uncertainty as how to deal with clients’ treatment needs. Clients were evasive, or requested specific drugs without presenting a reason, and came at a late stage for care and treatment. The main explanatory reason for these behaviours was believed to be the prevailing homosexual stigma in the Tanzanian context. While a relatively large body of research has established the negative effects of homosexual stigma on healthcare-seeking behaviours of MSM in Tanzania [20–22,42,43], there is limited research on the impact that stigma has on the provider’s assessment and treatment. In a qualitative study from Kenya, Micheni et al. revealed that healthcare providers experienced difficulties in engaging with MSM HIV patients due to the perceived (felt) stigma associated with homosexuality and HIV, which consequently impeded transparent interaction [44]. In addition, our data also indicated a sense of professional unpreparedness, a lack of skills to effectively communicate with MSM clients and challenges to connect with, and understand, their life issues. In Kenya, Taegtmeyer et al., identified service providers’ unfamiliarity with the MSM population as one of the key challenges for delivering HIV reduction counselling [45]. If pharmacy workers lack an understanding of MSM clients’ vulnerabilities to certain STIs and psychosocial issues, it might limit their capacity to provide valuable and accurate information, care and treatment.

It is possible that the reported lack of in-depth knowledge about the MSM community in this sample is one area where an injection of knowledge could address some of the problems related to assisting clients [46]. Evaluations from Kenya have shown that training concerning MSM’s sexual health could have a positive effect on healthcare workers’ preparedness in dealing with these issues [47]. Moreover, some participants in this study found the absence of a sensitised healthcare workforce problematic, as they were unable or reluctant to refer clients in need of diagnosis. They believed that the men’s fear of being judged and demeaned would deter them from seeking care from a non-sensitised provider. Trust between healthcare providers is an important feature for effective referral within the tiered healthcare system [48]. Healthcare workers, including pharmacists, must be assured that their clients or patients are treated properly and adequately at the place of referral. Our data, thus, shed light not only on the need of further sensitizing healthcare workers on MSM sexual health, but also on concerted efforts that improve coordination between providers engaged in MSM care, including staff from different disciplines (e.g. medicine, nursing, medical laboratory, as well as pharmacology).

### Methodological considerations

This study contributes with new knowledge about service provision to the MSM population among Tanzanian pharmacists and drugstore staff, with emphasis on perceptions and experiences. Several measures were taken to strengthen the study’s trustworthiness [49]. To enhance the credibility of the study, prior to data collection, the researchers engaged in a dialogue with key informants from the MSM community and health sector in order to gain an understanding of the cultural and social setting. Although each qualitative study to some extent is context-bound, the dependability and confirmability of the current study were enhanced by providing a detailed description of the methodological procedures, thereby enabling the reader to assess the research practices used [49]. Moreover, the reflective notes that were taken during the interviews helped the researchers to clarify any existing predisposition and reduced chances for bias during the analytical stage. Transferability of the findings might be limited, as the study was conducted in Dar es Salaam, and these findings might not be applicable in rural settings. However, given the prevailing hostility towards same-sex sexual practices in Sub-Saharan Africa and similar human and financial resource constraints in the healthcare system, the findings might be relevant in the broader perspective [10,50].

A potential limitation was the language barrier. Although English is commonly spoken, Swahili is the official national language in Tanzania. To facilitate communication with participants, an interpreter was always present during the interviews. While the interpreter was familiar with the topics discussed, there is a possibility that words and underlying meanings unintentionally were lost during the translation process in those instances where interpretation was needed (four interviews). To minimize the risk of such errors, after the interview, one of the researchers and the interpreter listened to the recording to confirm the accuracy of the translation, while at the same time comparing it with notes taken during the interview. Additionally, the surge of increasingly severe measures taken against the lesbian, gay, bisexual and transgender (LGBT) community in Tanzania during 2016 and 2017 [51,52] could have caused a change in the healthcare seeking behaviour among MSM. Consequently, the challenges that Tanzanian pharmacy workers experience when providing services to the MSM population might have changed since the data were collected.

## Conclusion

Our study revealed the perceived challenges that pharmacists and drugstore workers experienced when providing services to MSM clients in Dar es Salaam, Tanzania. As such, the study offers insights into a highly complex area of care and treatment by taking the perspective of the provider. Most pharmacy workers lacked sufficient skills to deal with MSM clients and their health needs and emphasised the need for further training but also more sensitised healthcare providers to whom they could refer clients to. In order to support the future generation of pharmacists in Tanzania, MSM’s sexual health issues should be integrated into the training curricula to better prepare pharmacists to deal with marginalised populations. Equally important is to offer interactive and continuous training to the broader professional group of pharmacy and drug store workers who lack formal pharmaceutical education. This training should ideally be integrated into already existing initiatives to ensure a systematic approach. Furthermore, inter-professional cooperation that harnesses MSM sensitised provider involvement from all tiers in the healthcare system is essential to offer complementary services in order to ensure proper STI care and treatment, which also has the potential to reduce unauthorised dispensing practices.

## Supplementary Material

Supplemental Material

Supplemental Material

## Paper context

Research suggests that pharmacy workers play a role in providing care and management of suspected STI symptoms among MSM in Tanzania due to the prevalent stigma of homosexuality. There is, however, limited research conducted on pharmacy workers as service-providers in relation to this population. Our research focuses on the challenges pharmacy workers’ encounter in providing STI services to MSM clients. Findings from this study could form basis for interventions targeting the health of stigmatised populations.
