# Supplementary material for: ‘Fighting an uphill battle’: a qualitative study of the challenges encountered by pharmacy workers when providing services to men who have sex with men in Dar es Salaam, Tanzania
Source: Glob Health Action. 2020 Jun 8;13(1):1770985. doi: 10.1080/16549716.2020.1770985 (PMC7448846; doi:10.1080/16549716.2020.1770985)
Supplement: Supplemental Material [file ZGHA_A_1770985_SM9037.docx]

**Interview guide- MSM and pharmacy services in Dar es Salaam**

**Introduction:**

“My name is ____.” We are currently undertake a smaller study that is part of a project called “The Spend Model Project”. The study is co-coordinated by the University of Dar es Salaam, Muhimbili University of Health and Allied Sciences and Lund University (Sweden).

The aim of the project is to address barriers related to sexual health services among men who have sex with men, MSM; and improve access to HIV and STI related services. This specific study is focusing particularly on the factors that might affect MSM to seek care at pharmacies for STI-related problems, and the experiences of the meeting with the pharmacist.

We hope that the findings of the study will contribute to an improved understanding of how MSM seek treatment, and how we can work in order to ensure safe and timely treatment for STI related problems.

I will ask some general questions about you and how you seek healthcare, but mostly about your views, opinions and experiences of using pharmacists and drug store workers as service providers when suffering from sexual health problems such as STIs.

If there are any other topics that you would like to raise relative to the main topics we discuss, please feel free to do so.

You may decline answers to any questions or discussion of any topics at any time.

The interview will take approximately one hour but if you have time constraints, I will make every effort to make our conversation shorter

I would like to make an audio recording of our conversation to be sure that I am able to capture your contribution in as much detail and as accurately as possible. I do not keep any identifying information about you personally, so there is no way to identify you in the study. If you prefer that I not record our conversation, I will only take written notes. In either case, our conversation is completely confidential. Please let me know what you are more comfortable with.

After we’ve spoken, if you have any further thoughts to share with the research time, please feel free to contact the principal investigator *(give card with contact details).*

**Verbal consent:** Before we move on, I will need to obtain your verbal consent to be interviewed. Would you like to participate in this interview?

**Questions**

**Opening question:**

I would like to begin by asking you to share a little about yourself.

How old are you?

Do you currently work or study? If yes, what do you study/where do you work?

How is you living situation? Alone, with family, friends?

**Healthcare services**

In Tanzania, as well as in other countries, sexually transmitted infections constitute a problem among same-sex practicing men. I would like to talk more about this issue and also remind you that everything you say to me is completely confidential and will not be linked back to you. But it is important knowledge for us in order to inform future interventions that aim to improve the sexual health among MSM.

1. First of all, how would you describe your existing knowledge regarding STIs?

*Probes:*

*Do you feel it is sufficient/insufficient? In what way?*

*Do you ever talk about STIs with your friends?*

2. When you need information about sexual health, such as safe sex, HIV, sexually transmitted infections, and so on, where do you seek this information?

Probe:

*Why would you seek there? Any specific reasons? In what ways does this information relate to your own health needs?*

3. Could you describe to me what are the most important things for you when you seek services related to you sexual health?

Probes:

*Confidentiality and privacy?*

*That the healthcare provider is knowledgeable?*

*Costs?*

*Availability of medicines?*

*The environment?*

4. As a MSM what are the unique healthcare needs that you have when it comes to STI?

5. When you suspect that you have a STI or any other kind of sexual health problem, what do you do?

As you know this study intends to focus on the role of pharmacy and drugstore workers as service providers and I would know like to talk about this.

6. Could you describe to me the first time you visited a pharmacy or drugstore for service provision regarding your sexual health- what made you take that decision?

Probe

*Was there a particular incident that affected you?*

*Did you friends talk about pharmacists and drugstore workers as sources of assistance?*

7. According to you, what are the main differences between how pharmacists and healthcare workers treat you as a MSM?

Probe

*Why do you think it is so?*

8. Could you describe your relationship with the pharmacist that you often go to?

Probe

*Are you close? In what way?*

*How does your relationship with the pharmacist affect your choice of care?*

*What made you choose him or her as a main provider?*

9. Have you disclosed your sexual orientation to the pharmacist that you often go to?

Probe

*If yes, describe to me when and how you did it?*

*What made you disclose it?*

*If no, why haven’t you disclosed it?*

10. Could you tell me about how the environment is at the pharmacy or drugstore where you usually go?

Probe

*Are there a lot of people?*

*Is it large or small?*

11. If you come to the pharmacy and there are a lot of people, what do you do?

Probe

*Do you stay and wait for your turn or do you leave?*

*What does the pharmacist do if he/she sees that you come and it’s crowded?*

12. What are the main challenges that you see with seeking assistance for STI-related problems at a pharmacy or drugstore?
